# Transient cell stiffening triggered by magnetic nanoparticle exposure

**DOI:** 10.1186/s12951-021-00790-y

**Published:** 2021-04-26

**Authors:** Jose E. Perez, Florian Fage, David Pereira, Ali Abou-Hassan, Sophie Asnacios, Atef Asnacios, Claire Wilhelm

**Affiliations:** 1 Laboratoire Matière et Systèmes Complexes MSC, UMR 7057, CNRS & University of Paris, 75205 Paris Cedex 13, France; 2 Institut Curie, Université PSL, Sorbonne Université, CNRS UMR 168, Laboratoire Physico Chimie Curie, 75005 Paris, France; 3 Sorbonne Université, CNRS UMR 8234, Physico-Chimie Des Électrolytes et Nanosystèmes InterfaciauX (PHENIX), 75005 Paris, France; 4 Faculty of Science and Engineering, UFR 925 Physics, Sorbonne Université, Paris, France

**Keywords:** Magnetic nanoparticles, Nano-bio interface, Single cell rheology, Parallel plate rheometer

## Abstract

**Background:**

The interactions between nanoparticles and the biological environment have long been studied, with toxicological assays being the most common experimental route. In parallel, recent growing evidence has brought into light the important role that cell mechanics play in numerous cell biological processes. However, despite the prevalence of nanotechnology applications in biology, and in particular the increased use of magnetic nanoparticles for cell therapy and imaging, the impact of nanoparticles on the cells’ mechanical properties remains poorly understood.

**Results:**

Here, we used a parallel plate rheometer to measure the impact of magnetic nanoparticles on the viscoelastic modulus G*(f) of individual cells. We show how the active uptake of nanoparticles translates into cell stiffening in a short time scale (< 30 min), at the single cell level. The cell stiffening effect is however less marked at the cell population level, when the cells are pre-labeled under a longer incubation time (2 h) with nanoparticles. 24 h later, the stiffening effect is no more present. Imaging of the nanoparticle uptake reveals almost immediate (within minutes) nanoparticle aggregation at the cell membrane, triggering early endocytosis, whereas nanoparticles are almost all confined in late or lysosomal endosomes after 2 h of uptake. Remarkably, this correlates well with the imaging of the actin cytoskeleton, with actin bundling being highly prevalent at early time points into the exposure to the nanoparticles, an effect that renormalizes after longer periods.

**Conclusions:**

Overall, this work evidences that magnetic nanoparticle internalization, coupled to cytoskeleton remodeling, contributes to a change in the cell mechanical properties within minutes of their initial contact, leading to an increase in cell rigidity. This effect appears to be transient, reduced after hours and disappearing 24 h after the internalization has taken place.
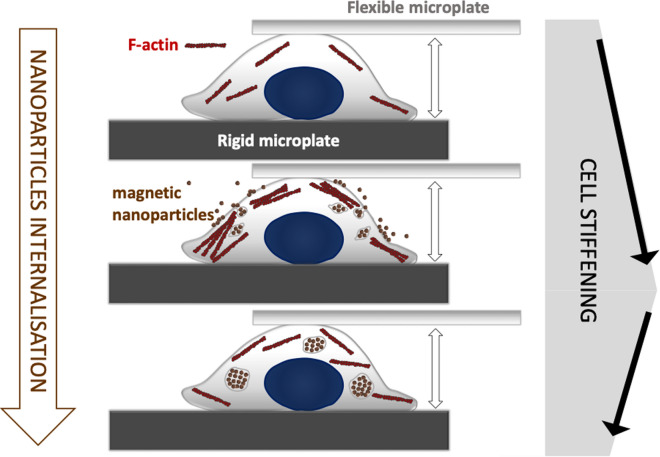

## Background

The rise of nanotechnology in many sectors including energy, environment and health has significantly increased the potential interactions between nanomaterials, human cells and their environment. It thus becomes mandatory to establish a better and almost complete understanding of the interface between nanoparticles and biological components, to provide in short to medium term predictive relationships between the functional activity of nanoparticles and their impact at the biological level [1]. This is one mission of the emerging nano-bio-interface field, and many advances have already been made towards the safe use of nanotechnologies by linking structural and physico-chemical characterizations of nanoparticles to their toxicity or biocompatibility.

For instance, the oxidative stress created by nanomaterials after cell contact is one pertinent readout towards a predictive paradigm in nanotoxicology. Nanoparticles made of oxides of manganese, chromium, nickel, or cobalt, for example, have proven to be toxic by generating significant oxidative stress [2–4]. By contrast, nanoparticles of iron oxides are very well tolerated, in vitro and in vivo, and are accepted as having low toxicity, hence their use in clinical imaging (MRI), hyperthermia and cancer cell therapy [5]. One other advantage of magnetic nanomaterials is their capacity for remote magnetic manipulation, enabling applications such as magnetic transfection, magnetogenetic manipulation and magnetic drug targeting [6–8]. In the field of regenerative medicine, magnetic nanomaterials have been envisaged as cell-specific MRI contrast agents to track implanted/injected therapeutic cells [9, 10], or to apply remote magnetic manipulations to retain magnetically-labeled cells at their site of in vivo implantation (e.g. the heart) [11, 12], or to be exploited for tissue engineering [13]. Ahead of these applications, many works have demonstrated that iron oxide nanoparticles can be efficiently taken up for instance by stem cells without affecting their function or their capacity for differentiation [14].

Among the test platforms to analyze the bio-physico-chemical interactions at the nano-bio-interface, the toxicological approach of the cellular impact of nanoparticles exposure is the most widespread [15]. Magnetic nanoparticles, for instance, have been shown to trigger a rearrangement of the actin cytoskeleton, to decrease the number of focal adhesion complexes and to slow the formation of long and extended microtubules [16–18]. Moreover, a possible oxidative stress toxicity related to the Fenton reaction and the initiation of ferroptosis mediated by Fe^2+^ ions should be noted [19, 20]. However, iron is present in state II only in magnetite, so that nanoparticles of iron oxide that have been fully oxidized to maghemite beforehand do not present this risk. Other factors, such as particle coating, can additionally translate to a decrease in cytotoxicity [21]. Nevertheless, it remains highly challenging to elucidate bio-physico-chemical interactions and the dynamic forces and molecular components that shape them, sometimes with not easily discernable cascading effects [22].

On the other hand, given recent advances in the understanding of the role of cell mechanics in many different biological processes, the evaluation of the possible adverse effects of nanoparticles on cell mechanical properties should perhaps be part of the standard methodology to assess the nano-bio interface [23]. Indeed, modern biophysics is more and more focused on the role of physical features and stimuli on cell functions, phenotypic orientation and differentiation. For example, the rigidity of the substrate or mechanical stresses such as cyclic ones can be sufficient to induce differentiation of multipotent or pluripotent stem cells, and local mechano-transduction can also induce differentiation [24]. Regardless, direct biophysical probing studies at the cell level of the impact of nanoparticle uptake on cells remains rare.

In the context of physical biosensing for cell functioning, the mechanics of cells themselves has been extensively studied. Owing to their cytoskeleton network and high water content conferring cells solid- and liquid-like behavior, they have been established as multi-scaled 3D viscoelastic active materials [25]. To study these cell mechanical properties, cutting-edge micro-rheological tools were developed to subject the cells to a controlled force or deformation, and measure the resulting deformation or force, respectively [26, 27]. Many of these micro-rheological techniques use probes at the subcellular level, such as atomic force microscopy (AFM) tips, optical or magnetic tweezers. The locality of the measurements is an asset to probe specific cell components, such as stress fibers, cortical actin, or the cytoplasm. By contrast, to address the impact of nanomaterials exposure, the mechanical measurement must be made at the cell level. This can be achieved by the use of a parallel plate rheometer, where a single cell is caught between two microplates, one being rigid, the other acting as a calibrated cantilever [28]. The whole cell’s rheological properties are then inferred. Importantly, and remarkably, despite variability in the absolute values of viscoelasticity provided, all methods converge towards the description of the cell rheology characterized by a power-law response [29], which reflects a broad distribution of relaxation times due to the multiscale architecture of the cell: the cell viscoelastic complex modulus G*(f) follows the power-law as a function of stimulation frequency *f*, G* = *G*_*0*_*exp(if)*^α^. The exponent α directly provides the signature of a liquid-like (α = 1) versus solid-like (α = 0) cell behavior [30].

How this robust cell micro-rheological behavior is affected by nanoparticles exposure still remains unknown. Here, due to their relevancy in the context of cancer therapy, imaging and regenerative medicine, we have selected the biocompatible iron oxide magnetic nanoparticles to address this question and to go beyond a potential direct toxicity and thus focus on the dynamical impact of nanoparticles on the cell mechanical properties. We hypothesized that the aforementioned effects that nanoparticles may have on the cytoskeleton might translate to a change in their viscoelastic properties, and used the parallel plate rheometer at the single cell level to explore this notion.

## Results and discussion

### Parallel plate rheometer

**Fig. 1:**
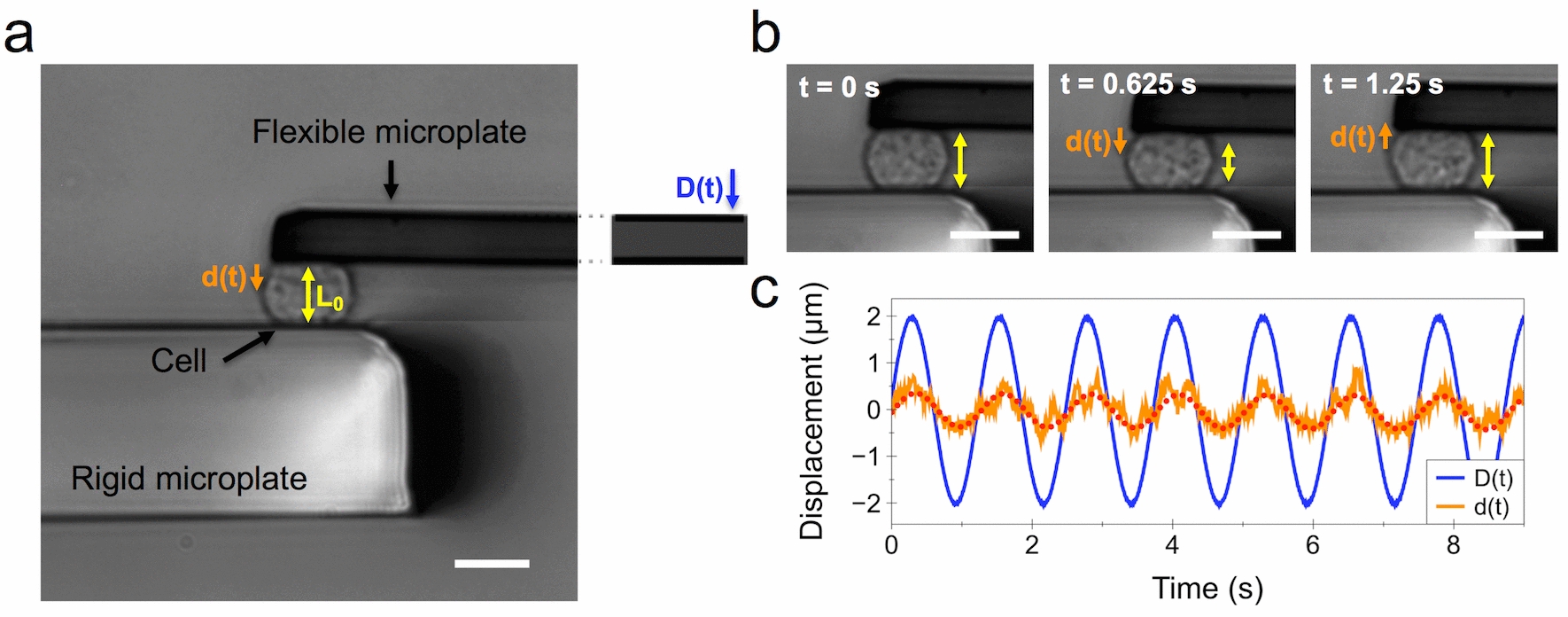
Parallel plate rheometer. **a** Rheometer consisting of a rigid and a flexible microplate, the latter being used as a spring of calibrated stiffness to apply a sinusoidal compression to an isolated single cell. A displacement *D(t)* is applied to the base of the flexible microplate, resulting in a displacement *d(t)* at its tip. **b** Illustration of sinusoidal cell compression over time at *f* = 0.8 Hz. **c** Visualization of the sinusoidal displacement *D(t)*, in blue, and the resulting displacement *d(t)*, in orange. The red-dotted line shows the sinusoidal fit of the resulting displacement *d(t)*. Scale bars = 10 µm

The effects of magnetic nanoparticles on the viscoelastic modulus G*(f) at the single cell level was assessed using a parallel plate rheometer [28, 31]. For this purpose, a single cell is caught between two glass microplates, one rigid and the other flexible (Fig. 1a). The flexible plate is used as a spring of calibrated stiffness, the deflection of which is proportional to the force applied to the cell. Typically, a sinusoidal displacement *D(t)* imposed to the base of the flexible microplate leads to a displacement *d(t)* at its tip (Fig. 1a). Since the rigid microplate is held at a constant position throughout the experiment, the displacement *d(t)* is equal to the cell deformation (elongation/shortening). The cell strain is thus obtained as *d(t)* over the cell’s unstrained length L_0_. The force applied to the cell, and thus the oscillating stress, is proportional to the flexible microplate deflection [*D(t)* − *d(t)*]. The relation between stress and strain is then used to calculate the viscoelastic modulus G*(f) (see “Methods” section). Figure 1b shows the bright field imaging of a cell and its deformation *d(t)* over the course of one cycle (*f* = 0.8 Hz). The typical sinusoidal displacement *D(t)* imposed at the base of the flexible microplate and the resulting cell deformation *d(t)* are shown in Fig. 1c. The dotted red line depicts the fit of *d(t)* used to obtain the complex modulus G*(f).

### Real-time monitoring of the mechanical response in single cells

**Fig. 2:**
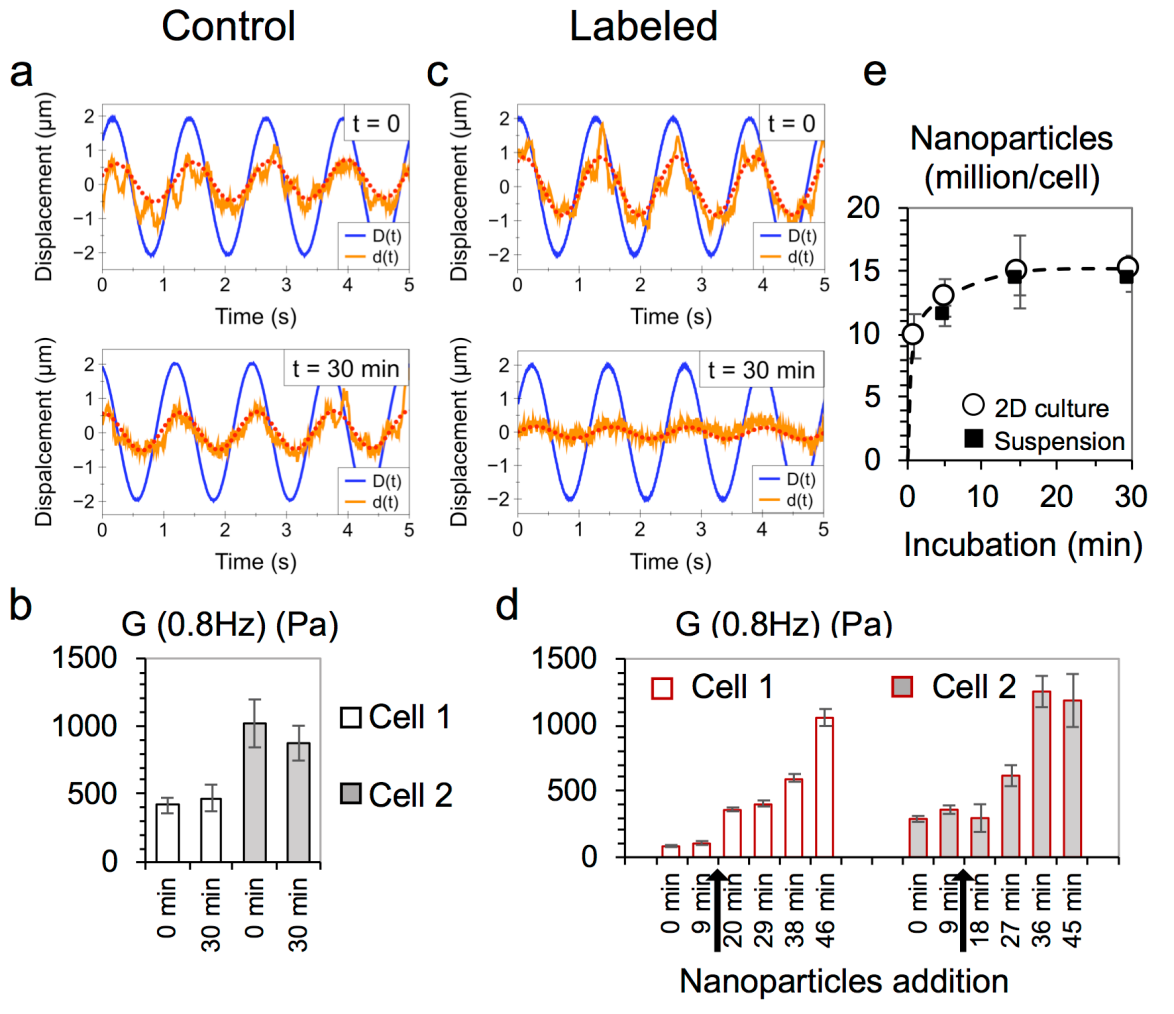
Real-time mechanical response in single cells. **a** Initial *D(t)* and resulting displacement *d(t)* of unlabeled control cells over a 30 min period, with fit of *d(t)* shown in red. **b** Viscoelastic modulus *G* (0.8 Hz) of two control cells after 30 min of measurement. **c** Initial *D(t)* and resulting displacement *d(t*) at the starting point of the measurement and after 30 min of magnetic nanoparticle exposure, with fit of *d(t)* shown in red. **d** Viscoelastic modulus *G* (0.8 Hz) of two labeled cells over 45 min of measurement. Black arrows signal the time of addition of nanoparticles. Data represent mean ± SEM (*n* = 3 measurements of the same cell over the shown timepoint). **e** Magnetic nanoparticle uptake at [Fe] = 50 mM up to 30 min of incubation for F9 cells labeled while in 2D culture or in suspension. Data represent mean ± SD (*n* = 50 cells analyzed for each time point)

Cancerous cells possess differential signatures in their mechanical properties depending on the cell type and level of malignancy, with changes in the cell cytoskeleton playing a key role in tumor progression [32, 33]. On the other hand, they are regularly exposed to magnetic nanoparticles in therapeutic applications, and are thus a good model to study changes in the mechanical response due to the nanoparticle-cell interactions. Here, we thus selected murine embryonal carcinoma F9 cells to unravel the impact that their interaction with magnetic nanoparticles may have at the level of cell mechanics. Similar evaluations are scarcely found in the literature. The parallel plate rheometer permits to catch one cell and then to measure its properties, in situ, thus being a unique platform to assess potential changes in the mechanical properties of one cell within the short timescale of the onset of nanoparticle internalization. Figure 2 shows the measured changes in the mechanical response in individual F9 cells, exposed or not to nanoparticles. An extracellular magnetic nanoparticle concentration of [Fe] = 50 mM was selected to trigger rapid cell interactions and discern a possible change in the cell viscoelasticity upon nanoparticle exposure. Those were added directly into the experimentation chamber of the parallel plate rheometer, and the viscoelastic modulus G*(f) was measured at 0.8 Hz frequency at set intervals before and after the addition of nanoparticles.

For the two control cells (no nanoparticles added during the course of the measurement), the typical displacement *d(t)* of the cell at the tip of the flexible microplate remains relatively unchanged (Fig. 2a), and the viscoelastic modulus G* (0.8 Hz) is similar over a 30 min measurement time (Fig. 2b). On the other hand, the cells subjected to the magnetic nanoparticles labeling condition showed a decreased cell deformation *d(t)* (Fig. 2c), corresponding to an increase in their viscoelastic modulus G* (0.8 Hz) up to 30 min after labeling, at tenfold and fourfold for cell 1 and cell 2, respectively (Fig. 2d). This is consistent with the quantification of nanoparticle uptake by single cell magnetophoresis over the same time interval for this labeling condition, performed with F9 cells in suspension to mimic the conditions of the experimentation chamber of the parallel plate rheometer. The uptake of nanoparticles is a rather fast process, with internalization taking place within minutes: each cell takes up on average 11.4 ± 0.8 million NPs after 5 min of incubation and up to 14.2 ± 1.1 million after 30 min (Fig. 2e). Interestingly, F9 cells labeled while in typical 2D culture showed very similar nanoparticle uptake (Fig. 2e). Overall, these measurements at the single cell level suggest that the interactions of magnetic nanoparticles with cells triggers a stiffening response within the first 30 min after their initial interfacing.

As previously mentioned, there are only a few studies found in the literature which focus on the impact of nanoparticles on the mechanical properties of cells, especially in the short timescale following cell-nanoparticle contact. Tay et. al showed that labeling of TR146 epithelial cells with either SiO_2_ or TiO_2_ nanoparticles increased the cells’ contractility (which is related to rigidity [34]) within 30 min, increasing significantly after the first hours and impairing cell migration [35]. This was attributed to a nanoparticle-mediated disruption of the microtubules assembly. Here, a similar stiffening effect is observed at the single cell level within the same time period. Whether this cell stiffening would carry over after a more prolonged incubation time with nanoparticles was next investigated, this time at the cell population level.

### Impact of nanoparticles exposure on cell viscoelasticity at the cell population level

**Fig. 3:**
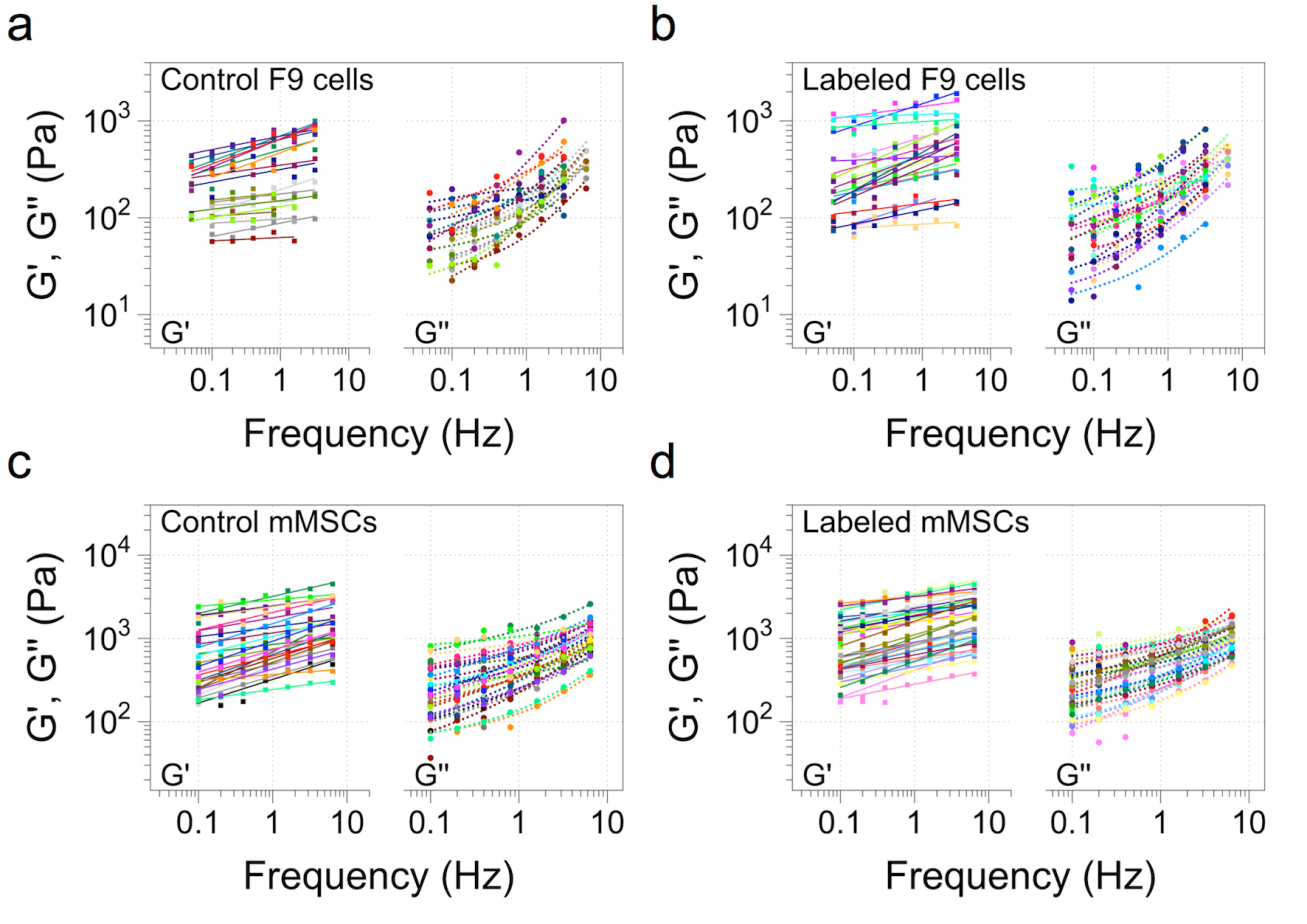
Elastic (*G’*) and viscous (*G’’*) moduli of F9 and mMSCs as a function of the frequency. **a**, **b** Control and labeled F9 cells, respectively. **c**, **d** Control and labeled mMSCs, respectively. The cells were labeled with magnetic nanoparticles at [Fe] = 4 mM for 2 h before analysis. Each line of the elastic and viscous moduli corresponds to one cell analyzed. (*n* = 23 for F9 control cells; *n* = 24 for F9 labeled cells; *n* = 29 for control mMSCs; *n* = 34 for labeled mMSCs)

In all diagnosis and therapeutic applications involving the internalization of magnetic nanoparticles by cells, these are generally taken up in 2D culture. Then, the cells are detached and used for imaging, tissue engineering or for a specific therapy. The correct experimental set-up to assess the mechanical impact of nanoparticles on cells thus appears to be at the cell population level, for control unlabeled cells and labeled ones, under a pre-labeling condition of a set time of incubation in order to mimic these routine clinical and imaging conditions. A second cell line, murine mesenchymal stem cells (mMSCs), was selected for their relevance in the regenerative medicine field. Besides, these cells exhibit a significantly higher viscoelasticity modulus than the F9 cells, and hence are also interesting to attest if the initial value of viscoelasticity would translate to a differential response when labeled with magnetic nanoparticles. The labeling condition was set to an incubation at [Fe] = 4 mM for 2 h for both cell types, performed in the corresponding culture flask, which translated to an iron load of 10.8 ± 4 and 23.3 ± 5.9 million NPs/cell for F9 and mMSCs, respectively, as quantified through magnetophoresis (Additional file 1: Figure S1). Over 20 individual cells were analyzed right after labeling and detachment from the culture flask using the parallel plate rheometer. Figure 3 shows the elastic [*G’ (f)*] and viscous [*G’’ (f)*] moduli for the two cell types, and for unlabeled (control) and magnetically-labeled populations, as a function of increasing frequency.

These data show that the complex viscoelastic modulus G*(f) follows a power-law as a function of frequency for both cell types, as previously reported for other cell types [29, 36]. Besides, it evidences that the labeling with nanoparticles does not impact this power-law rheology. Indeed, each line plotted in Fig. 3 corresponds to the fit to the power-law for both *G’(f)* and *G’’ (f)* with the same exponent α (see “Methods” section). The viscous modulus *G’’ (f)* was approximately three times lower than the elastic one, for both cell lines and in both control and labeled conditions. The exponent α is correlated with possessing a solid-like (α = 0) or liquid-like (α = 1) behavior [30]. Here, it was systematically found to in the ~ 0.2 range, revealing cells to be more elastic than viscous. The power-law behavior of the viscoelastic modulus G*(f) is thus very robust, and is conserved for the two cell lines tested after magnetic labeling at the selected dose.

**Fig. 4:**
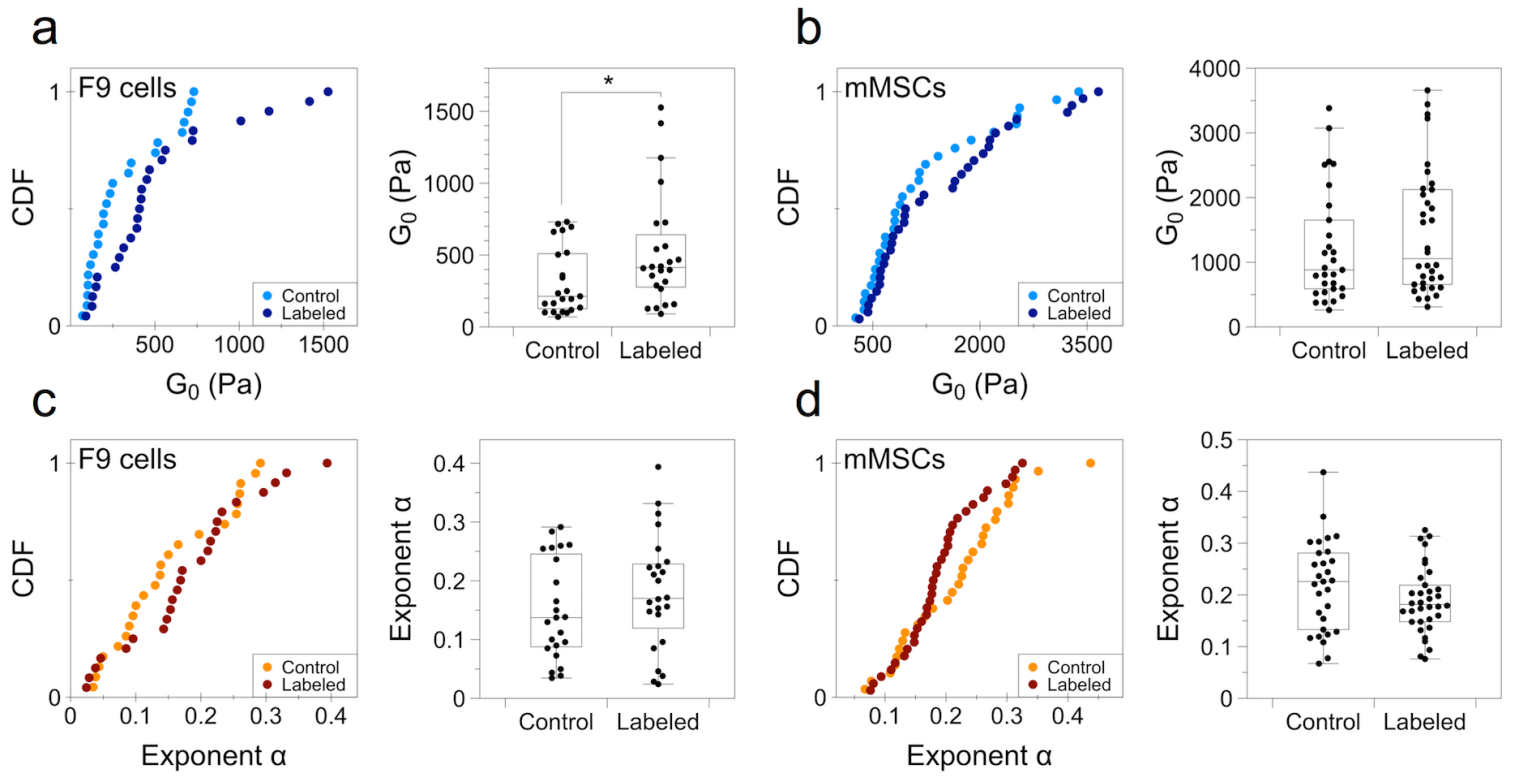
Cumulative distribution function (CDF) and box plot representation of the prefactor *G*_*0*_ and exponent α. **a**, **b** Prefactor G_0_ for F9 cells and mMSCs, respectively. **c**, **d** Exponent α for F9 cells and mMSCs, respectively. (*n* = 23 for F9 control cells; *n* = 24 for F9 labeled cells; *n* = 29 for control mMSCs; *n* = 34 for labeled mMSCs; *p < 0.05)

Additionally, using the power-law analysis of each cell population, the prefactor *G*_*0*_ can also be extracted. As previously mentioned, the exponent α is closer to the solid-like situation, and as a result the cells show a more elastic than viscous behavior. Thus, the prefactor *G*_*0*_ is mostly dominated by the *G’* modulus. Figure 4 thus shows the cumulative distribution and box plot representation of the power-law prefactor *G*_*0*_ and exponent α for both F9 cells and mMSCs. For comparison with the individual cell experiment (Fig. 2), the distribution of *G*’ (0.8 Hz) is provided in the Additional file 1: Figure S2. The F9 cells show a lower overall median *G*_*0*_ compared to mMSCs, at 213 ± 44 and 882 ± 124 Pa, respectively (Fig. 4a, b). The uptake of magnetic nanoparticles under the analyzed labeling condition ([Fe] = 4 mM for 2 h of incubation) led to a twofold and a 1.2-fold increase in the median of the prefactor *G*_*0*_ for F9 and mMSCs, respectively, and being significant only for the former. The exponent α did not change significantly for either cell line, with an average mean of 0.15 ± 0.08 and 0.17 ± 0.08 for control and labeled F9 cells, respectively, and of 0.21 ± 0.08 and 0.19 ± 0.06 for control and labeled mMSCs, respectively (Fig. 4c, d). Noteworthy, in the box plot distribution, a noticeable trend can be seen for mMSCs, with labeled cells appearing more elastic than non-labeled ones, though in a not significant manner. Importantly, the significant increase observed in the prefactor *G*_*0*_ for labeled F9 cells renormalized when the cells were analyzed 24 h later, with very similar cumulative distributions for both control and labeled cells (Additional file 1: Figure S3). For this condition, the 2 h incubation condition at [Fe] = 4 mM was conserved, then the nanoparticles were removed and the cells were placed in complete medium and left to grow under normal conditions for 24 h before analysis.

Other works reporting nanoparticle-induced changes in the mechanical properties of cells within time frames of exposure similar to those reported here have so far studied different composition nanomaterials, and used AFM to probe such interactions. For instance, in agreement with our results, the cell rigidity of human MSCs was found to be more than twofold in cells treated with SiO_2_ nanoparticles compared to the control within the first hour of treatment, an effect which later decreased to approximately 1.6-fold after 24 h [37]. However, the same nanoparticles interfaced with human aortic endothelial cells for 4 h, yet at a lower dose, did not trigger any significant changes in the cells’ mechanical properties [38]. By contrast, within the same latter work, ZnO nanoparticles led again to a cell stiffening effect. Later on, the same group reported cell stiffening at low uptake levels of Ag nanoparticles in alveolar macrophages, which then translated to cell softening for high uptake levels, probably due to cell toxicity and damage [39]. Lastly, in the one study using magnetic nanoparticles, porcine aortic endothelial cells cultured with uncoated iron oxide nanoparticles for 24 h showed a 1.5-fold increase in their elastic modulus compared to the untreated control. In this case however, the long incubation period lead to the production of cytotoxic reactive oxygen species.

A common universality found in the increased cell rigidity after interactions with these diverse types of nanoparticles is the rearrangement and alterations of the cell cytoskeleton. Indeed, and in the context of the mechanical properties of mammalian cells, it is known that the actin cytoskeleton exhibits a viscoelastic behavior and is thus one of the main contributors to the cell’s viscoelastic profile [40, 41], insofar as being able to recapitulate in vitro the mechanical properties of cells when cross-linked with the actin-binding protein filamin A [42]. The microtubules too play a role in balancing the cytoskeleton tensile stress, which is proportional to the cell stiffness [43, 44], and their depolymerization has been linked to cell contraction [45]. In line with this, magnetic nanoparticles have also been linked to cytoskeleton alterations. The incubation of human umbilical vein endothelial cells with citrate- and dextran-coated iron oxide nanoparticles showed a thinner, disorganized actin filament network and irregularities in their morphology, as well as the disruption of the microtubule network, possibly due to polymerization interference [17]. The iron oxide contrast agent formulation Resovist, as well as magnetoliposomes, were both additionally found to alter the structural organization of actin and induced a more compact microtubule network in human blood outgrowth endothelial cells [18].

In context with the active role of actin in the cell mechanical properties, we therefore decided to observe the organization of this cytoskeleton component under our experimental conditions to discern a possible correlation with the reported increase in cell stiffening. Additionally, we imaged the nanoparticle uptake process, given the role of the actin cytoskeleton in the endocytosis pathway [46].

### Imaging of the magnetic nanoparticle-cell interactions and the actin cytoskeleton

**Fig. 5:**
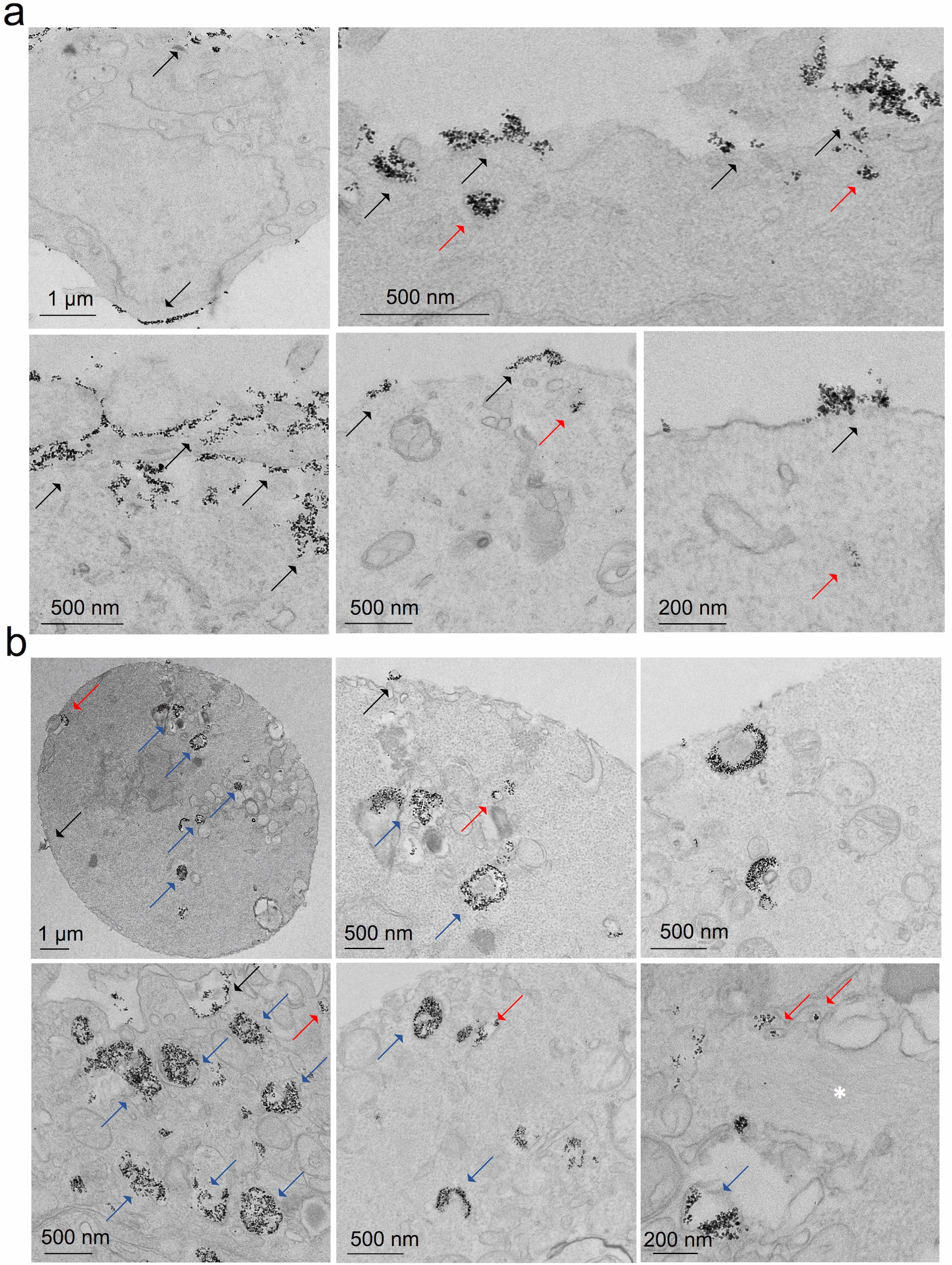
TEM imaging of F9 cells labeled with magnetic nanoparticles. **a** Cells incubated with nanoparticles at [Fe] = 50 mM for 5 min incubation. **b** Cells labeled at [Fe] = 4 mM for 2 h incubation. Black arrows show nanoparticles accumulated near the cell membrane, whereas red and blue ones depict those located in early and late or lysosomal endosomes, respectively

In order to corroborate that the increase in cell stiffness observed for F9 cells labeled with magnetic nanoparticles correlated with the internalization of nanoparticles under very short time labeling conditions, the uptake process was observed at the nanoscale by transmission electron microscopy (TEM) imaging. Both the high-dose, short labeling condition, chosen here at 5 min incubation, and the longer one of 2 h used for the probing of the cell population were investigated. Typical images for these two conditions are shown in Fig. 5, and additional panels of images are shown in the Additional file 1: Figure S4 (5 min) and Figure S5 (2 h). For the 5 min incubation condition, nanoparticles showed as aggregates near the cell membrane and in early endosomes (Fig. 5a). By contrast, for the 2 h of incubation condition, magnetic nanoparticles can be observed either in the vicinity of the cell membrane, early endosomes and late or lysosomal endosomes (Fig. 5b).

**Fig. 6:**
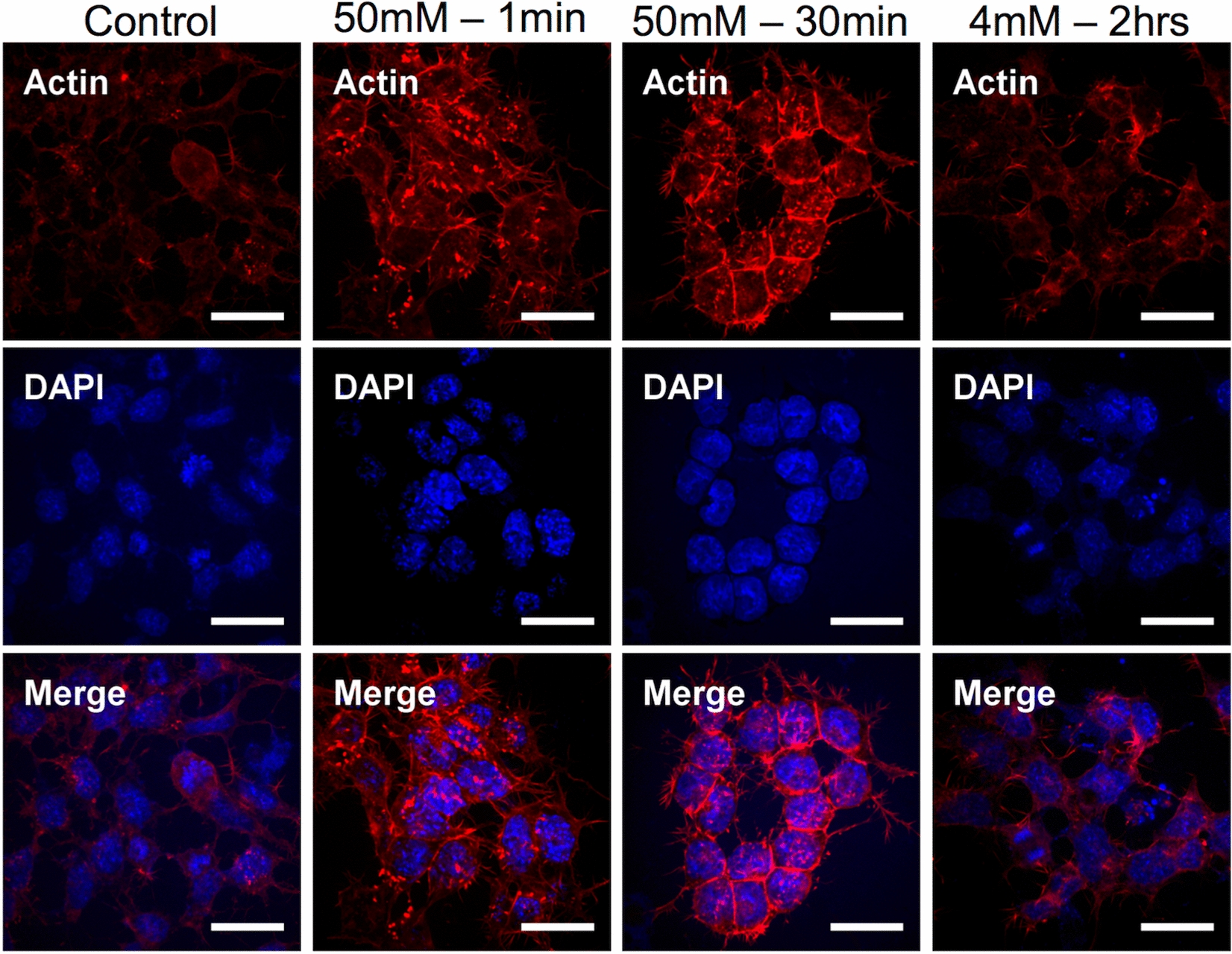
Fluorescent staining of actin filaments observed under confocal microscopy. F9 cells incubated with magnetic nanoparticles under three incubation conditions and stained for actin (red) and cell nuclei (DAPI). Actin bundling (bright red fluorescent spots) can be observed in the cell cytoplasm and cell membrane for the [Fe] = 50 mM condition. Scale bars = 30 µm

We further observed the organization of the actin filaments under similar labeling conditions through fluorescence microscopy, with representative images shown in Fig. 6. For the [Fe] = 50 mM magnetic nanoparticles condition at 1 min of incubation, a quite sharp increase in actin bundling can be observed, which is further translated to actin accumulation at the membrane of the cells after 30 min of incubation. Interestingly, at the 2 h incubation at [Fe] = 4 mM the expression of actin resembles more closely that of the control cells, with fewer actin bundles present. Additional images under the same conditions are provided in Additional file 1: Figure S6. Overall, the actin cytoskeleton response is time and concentration dependent, yet its restructuring appears to be transient too, resembling that of control cells after 2 h of incubation.

The nanoparticle intracellular uptake imaging highlights differences between the two incubation conditions. At short incubation times, the majority of nanoparticles are localized on the cell membrane and early endosomes, suggesting an active and ongoing uptake. 2 h later, most of the nanoparticles are already localized in late or lysosomal endosomes, thus close to the final endocytosis stages. These observations are in line with the increased F-actin activity detected at short times. The F-actin increase correlates with the onset of the rapid cell stiffening within the first 30 min of interaction with the nanoparticles (Fig. 2), thus probably translating or contributing to the sudden change in the cells’ viscoelastic properties. The renormalization in F-actin activity observed after 2 h further correlates with the less marked stiffening for this incubation condition, though it was still significant compared to the control (Fig. 4). These results highlight a possible dose-dependent effect of the magnetic nanoparticles on the cell mechanical properties, with higher loads translating to a more pronounced cytoskeleton rearrangement and cell stiffening. As discussed before, the changes in F-actin are in agreement with those previously reported for iron oxide nanoparticles and other tested materials.

Frequency-dependent rheological measurements directly reflect the molecular changes of the cytoskeleton, as recently confirmed in the high-frequency range (100 Hz–1 kHz) [47]. Cell stiffening is of particular relevance in cancer research, as cancerous cell lines are typically regarded to be softer than their benign counterparts [48, 49], a quality also correlated to metastatic potential [50]. A stiffening of cancer cells mediated by nanoparticles could thus have relevance in cancer therapy, and would be very worth investigating regarding a possible effect on invasion and metastatic potential. A possible route would then be to make the stiffening effect less transient, for instance by stopping the nanoparticles internalization process at the first membrane-bound events of endocytosis.

## Conclusion

In summary, we show that magnetic nanoparticle cell uptake takes place within minutes, and that its onset initiates a mechanical response in the cells that translates into an increased rigidity in this short timescale. This response is probably associated to a cytoskeleton remodeling, as suggested by the increased presence of actin fibers in the cell cytoplasm and cell membrane, acting in part as a driving force of nanoparticle internalization upon contact with the cell membrane. Moreover, the increase in rigidity is less pronounced after full internalization of the nanoparticles has taken place, with them being mostly found in late or lysosomal endosomes, and after 24 h the effect is no more present, with cell viscoelasticity being perfectly similar to that of the control. We believe this work and analysis at the single cell level has implications in the full understanding of the role of nanoparticle cell-interactions in the mechanical properties of cells, where experimental evaluation remains scarce. In particular, it evidences a strong and almost immediate response upon nanoparticle exposure, with cell rigidity being more than doubled within minutes, then renormalized hours later.

## Methods

### Iron oxide nanoparticles

Iron oxide nanoparticles were synthesized using the Massart procedure of iron salts co-precipitation [51]. The resulting magnetite (Fe_3_O_4_) nanoparticles were oxidized into maghemite (γ-Fe_2_O_3_) and then stabilized in aqueous solution via citrate chelation. The resulting nanoparticles possess an average diameter of 8 nm. When suspended in RPMI cell medium supplemented with 5 mM citrate to ensure colloidal stability, the initial hydrodynamic diameter of the nanoparticles was of 49.5 nm, and of 52.3 and 55.1 nm after 15 and 30 min, respectively, as measured by Nanosizer (Malvern Panalytical) (Additional file 1: Figure S7).

### Cell culture

F9 murine embryonal carcinoma cells (ATCC) and murine mesenchymal stem cells were cultured in Dulbecco’s Modified Eagle Medium (Gibco) supplemented with 10% fetal bovine serum (Gibco) and 1% penicillin–streptomycin (Gibco) and grown at 37 °C in a humidified incubator with 5% CO_2_. The cells were detached with 0.05% trypsin–EDTA (Gibco) after reaching the desired confluence.

### Cell labeling and magnetic nanoparticle internalization quantification

The cells were labeled under two different conditions. The first one consisted of a solution of magnetic nanoparticles at [Fe] = 4 mM suspended in RPMI cell medium without serum and supplemented with citrate at 5 mM to ensure colloidal stability, and left to incubate for 2 h. The second one was of [Fe] = 50 mM stabilized with citrate at 25 mM and suspended in cell medium (2% fetal bovine serum). The iron uptake per cell was calculated through single cell magnetophoresis [52]. Briefly, the magnetization of a single cell, *M*_*cell*_, is obtained from balancing its drag force *F*_*d*_ = *3πηDν* to the magnetic force *F*_*m*_ = *M*_cell_*gradB as it is attracted and moved by a constant magnetic gradient. The magnetization of a single cell thus yields the total amount of iron uptake.

### Parallel plate rheometer

The viscoelastic complex modulus at the single cell level was measured using a parallel plate rheometer, described in full detail elsewhere [28, 31]. In summary, it consists of two glass microplates, one rigid and one flexible. The flexible microplates used in this work were calibrated with respect to a standard one to obtain their respective stiffness *k*, and all microplates were cleaned in piranha solution (67% sulfuric acid and 33% hydrogen peroxide) for 15 min and then rinsed thoroughly with deionized water before experiments were conducted. The rigid and flexible microplates were mounted on two rotating arms fixed symmetrically on each side of the optical axis of a Leica DMIRB optical microscope (Leica Microsystems), and coupled to a piezoelectric *x, y, z* stage (Polytech-PI) supported by a *x, y, z* micrometer-driven stage (OptoSigma, Photonetics). This arrangement permits manual and computer-controlled precise displacement of both microplates. The whole setup is enclosed in a Plexiglas chamber maintained at 37 °C by an Air-Therm heater controller (World Precision Instruments) and placed on a TS-150 vibration isolation table (HWL Scientific Instruments).

Bright field imaging of the cells in the rheometer was performed using a 63 × objective and a Lumenera Infinity 3–6 CCD camera (Lumenera Corporation). A S3969 position-sensitive detector was used to track the displacement of the flexible microplate, the output signal of which was acquired by a PCI-6035E data acquisition board and processed with the LabView software.

All single cell manipulations were performed in the experimentation chamber of the parallel plate rheometer filled with culture medium supplemented with serum. The cells were analyzed in suspension (after detachment from the culture flask) for all the experiments shown.

### Viscoelastic modulus of a single cell

The mechanical response of each cell, i.e. its viscoelastic modulus G*(f), is determined by the ratio between the amplitude of the oscillating stress *σ(t)* and that of the resulting strain *ε(t)*, as well as the phase shift ϕ between the two signals. Assuming a sinusoidal displacement *D(t)* applied at the base of the flexible microplate (Fig. 1):

1 $$D\left(t\right)={D}_{0}\,{\text{exp}}\left(i2\pi ft\right),$$

The displacement at the tip of the microplate *d(t)* is then sinusoidal with the same frequency *f* as *D(t)* but with a phase shift ϕ:

2 $$d\left(t\right)={d}_{0}\,{\text{exp}}[i\left(2\pi ft+ \phi \right)].$$

Then, we can define the stress *σ(t)* in terms of the flexible microplate deflection:

3 $$\begin{aligned}\sigma \left(t\right)& =\frac{F\left(t\right)}{S}=\frac{k\left[D\left(t\right)-d\left(t\right)\right]}{S} \\ & \quad =\frac{k}{S}{\text{exp}}\left(i2\pi ft\right)[{D}_{0}-{d}_{0}\,{\text{exp}}\left(i\phi \right],\end{aligned}$$

where *F* is the force applied to the cell, *S* the area of contact between the cell and the microplates and *k* the flexible microplate stiffness. Consequently, the strain *ε(t)* is defined as the cell deformation, equal to *d(t)*, divided by the initial unstrained cell length *L*_*0*_:

4 $$\varepsilon \left(t\right)=\frac{{d}_{0}}{{L}_{0}}{\text{exp}}[i\left(2\pi ft+ \phi \right)].$$

Thus, the viscoelastic modulus G*(f), is the ratio of σ(t)/ε(t):

5 $${G}^{*}\left(f\right)=\frac{k{L}_{0}}{S}\left[\frac{{D}_{0}}{{d}_{0}}\text{exp}\left(-i\phi \right)-1\right],$$

where the storage or elastic modulus G’ (*f*) corresponds to the real part:

6 $${G}^{\prime} \left(f\right)=\frac{k{L}_{0}}{S}\left[\frac{{D}_{0}}{{d}_{0}}{\cos}(\phi )-1\right],$$

whereas the loss or viscous modulus G’’ (*f*) is the imaginary one:

7 $${G}^{\prime\prime} \left(f\right)=-\frac{k{L}_{0}}{S}\frac{{D}_{0}}{{d}_{0}}{\sin}(\phi ).$$

All calculations of *G’ (f)* and *G’’ (f)* were computed using the Matlab software (Mathworks) using an in-house program. The prefactor *G*_*0*_ was obtained from the exhibited power-law behavior of the viscoelastic modulus G*(f), taking into account structural damping [29], all fitted with the Matlab software:

8 $${G}^{\prime}={G}_{0}^{\prime}{f}^{\alpha }\, and\, {G}^{\prime\prime}={G}_{0}^{\prime\prime}{f}^{\alpha }+2\pi \eta f,$$

where *η* is the damping coefficient, and from which:

9 $${G}_{0}=\sqrt{{\left({G}_{0}^{\prime}\right)}^{2}+{\left({G}_{0}^{\prime\prime}\right)}^{2}}$$

### Transmission electron microscopy imaging

F9 cells were labeled with magnetic nanoparticles and then detached using 0.05% trypsin–EDTA and pelleted under centrifugation. Then, cells were fixed in 2.5% glutaraldehyde in 0.1 M cacodylate buffer at pH 7.2 for 1 h at room temperature and then resuspended in cacodylate buffer. The cell pellets were then contrasted with Oolong Tea Extract (OTE) cacodylate buffer, post-fixed with 1% osmium tetroxide and 1.5% potassium cyanoferrate, and dehydrated in ethanol baths from 30 to 100% before embedding in epoxy resins. Ultrathin sections of 70 nm were finally placed on copper grids and examined with a Hitachi HT 7700 transmission electron microscope operating at 80 kV.

### Actin fluorescent staining

After labeling with magnetic nanoparticles under the desired conditions, F9 cells grown in glass coverslips were fixed in 4% paraformaldehyde solution for 1 h at room temperature. The cells were then washed with phosphate buffered saline (PBS) and then stained using the SPY555-actin (SC202, Spirochrome) fluorescent dye at a 1:1000 dilution in PBS for 1 h at room temperature. Then, the cells were washed with PBS and the cell nuclei were stained with DAPI (D9564, Sigma-Aldrich) at a 1:1000 dilution in PBS for 30 min at room temperature. After washing, samples were mounted on microscope slides using Fluoromount Aqueous Mounting Medium (F4680, Sigma-Aldrich). After gelation of the mounting medium, fluorescence imaging was performed with an Olympus IX81F-3 inverted microscope (Olympus) coupled with a laser dual spinning disc unit (Yokogawa CSU-X1) and an Andor iXon^EM^ + CDD camera (Andor Technology), using a 60 × oil immersion objective. Images were processed using the ImageJ software.

### Statistical analysis

Due to the complex G* modulus following a log-normal distribution, as confirmed through the Anderson–Darling test using the Matlab software, the average values reported throughout the manuscript correspond to the median of the single cell measurements of the given two cell populations, with the error corresponding to the standard deviation of the mean of the log-normal distribution. Statistical analysis was performed using a Two-sample t-test of the log-normal distributions, with a statistical significance considered for values of *p* < 0.05 vs. the specific control. For the exponent α, the average values reported correspond to the geometric mean, whereas the error is the standard deviation of the mean.

## Supplementary Information

**Additional file 1: Figure S1.** Magnetophoresis quantification of iron uptake. **a** and **b** Distribution of the uptake of iron nanoparticles in F9 cells and mMSCs, respectively. Incubation condition was of [Fe] = 4 mM for 2 h. (*n* = 200 for F9 control cells; *n* = 234 for F9 labeled cells). **Figure S2.** Cumulative distribution function (CDF) and box plot representation of G’ (0.8 Hz) for a F9 cells and b mMSCs. (n = 23 for F9 control cells; n = 24 for F9 labeled cells; n = 29 for control mMSCs; n = 34 for labeled mMSCs; *p < 0.05). **Figure S3.** Viscoelastic modulus of F9 cells analyzed 24 h after labeling with magnetic nanoparticles at [Fe] = 4 mM during a 2 h incubation. **a** and **b** Elastic (*G’*) and viscous (*G’’*) moduli as a function of oscillating stress for control and labeled cells, respectively. **c**, **d** and **e** Cumulative distribution function (CDF) and box plot representation of the prefactor *G*_*0*_, exponent α and *G’* (0.8 Hz), respectively. (n = 14 for control cells; n = 18 for labeled cells; *p < 0.05). **Figure S4.** TEM imaging of F9 cells labeled with magnetic nanoparticles at [Fe] = 50 mM during a 5 min incubation. **Figure S5.** TEM imaging of F9 cells labeled with magnetic nanoparticles at [Fe] = 2 mM during a 2 h incubation. **Figure S6.** Fluorescent staining of actin filaments observed under confocal microscopy. F9 cells incubated with magnetic nanoparticles under three incubation conditions and stained for actin (red) and cell nuclei (DAPI). Scale bars = 30 µm. **Figure S7.** Evolution of the hydrodynamic size of the nanoparticles when dispersed in RPMI cell medium supplemented with 5 mM of citrate, as measured by Nanosizer (size distribution by intensity). It evidences the good colloidal stability of the nanoparticles. On average, the hydrodynamic diameter equals 49.5 nm initially, and 52.3 and 55.1 nm after 15 and 30 min, respectively.

## Data Availability

The datasets used and analyzed for this work are available upon request to the corresponding author.
