# Supplementary material for: Transient cell stiffening triggered by magnetic nanoparticle exposure
Source: J Nanobiotechnology. 2021 Apr 26;19:117. doi: 10.1186/s12951-021-00790-y (PMC8074464; doi:10.1186/s12951-021-00790-y)
Supplement: Supplementary file 1 — Additional file 1: Figure S1. Magnetophoresis quantification of iron uptake. a and b Distribution of the uptake of iron nanoparticles in F9 cells and mMSCs, respectively. Incubation condition was of [Fe] = 4 mM for 2 h. (n = 200 for F9 control cells; n = 234 for F9 labeled cells). Figure S2. Cumulative distribution function (CDF) and box plot representation of G’ (0.8 Hz) for a F9 cells and b mMSCs. (n = 23 for F9 control cells; n = 24 for F9 labeled cells; n = 29 for control mMSCs; n = 34 for labeled mMSCs; *p < 0.05). Figure S3. Viscoelastic modulus of F9 cells analyzed 24 h after labeling with magnetic nanoparticles at [Fe] = 4 mM during a 2 h incubation. a and b Elastic (G’) and viscous (G’’) moduli as a function of oscillating stress for control and labeled cells, respectively. c, d and e Cumulative distribution function (CDF) and box plot representation of the prefactor G0, exponent α and G’ (0.8 Hz), respectively. (n = 14 for control cells; n = 18 for labeled cells; *p < 0.05). Figure S4. TEM imaging of F9 cells labeled with magnetic nanoparticles at [Fe] = 50 mM during a 5 min incubation. Figure S5. TEM imaging of F9 cells labeled with magnetic nanoparticles at [Fe] = 2 mM during a 2 h incubation. Figure S6. Fluorescent staining of actin filaments observed under confocal microscopy. F9 cells incubated with magnetic nanoparticles under three incubation conditions and stained for actin (red) and cell nuclei (DAPI). Scale bars = 30 µm. Figure S7. Evolution of the hydrodynamic size of the nanoparticles when dispersed in RPMI cell medium supplemented with 5 mM of citrate, as measured by Nanosizer (size distribution by intensity). It evidences the good colloidal stability of the nanoparticles. On average, the hydrodynamic diameter equals 49.5 nm initially, and 52.3 and 55.1 nm after 15 and 30 min, respectively. [file 12951_2021_790_MOESM1_ESM.docx]

**Additional Information**


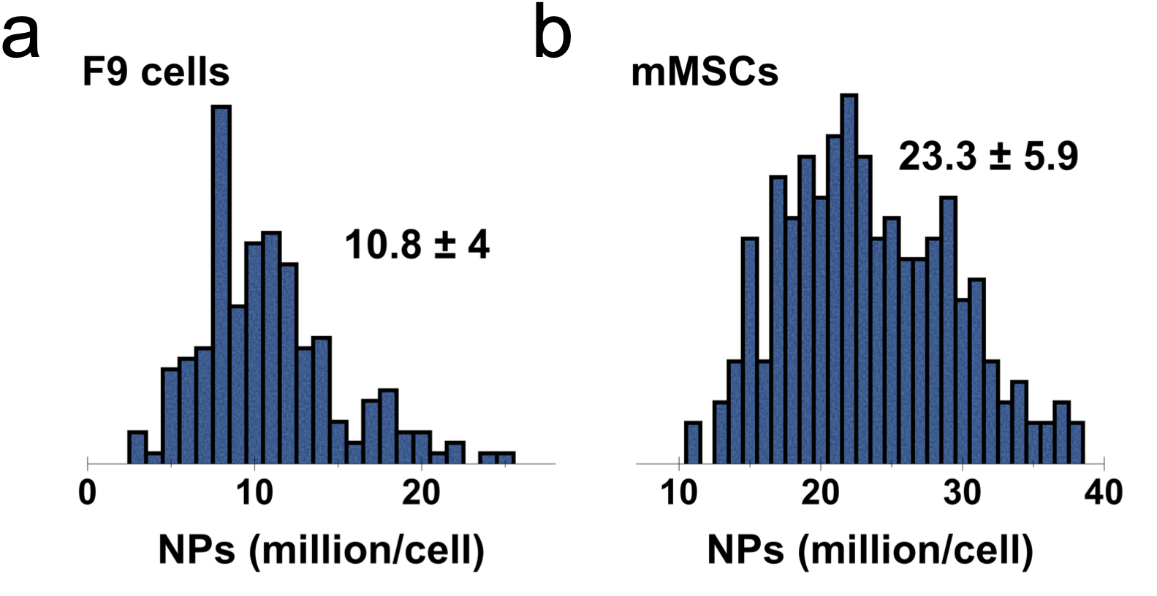


**Figure S1.** Magnetophoresis quantification of iron uptake. **a** and **b** Distribution of the uptake of iron nanoparticles in F9 cells and mMSCs, respectively. Incubation condition was of [Fe] = 4 mM for 2 hours. (*n* = 200 for F9 control cells; *n* = 234 for F9 labeled cells).


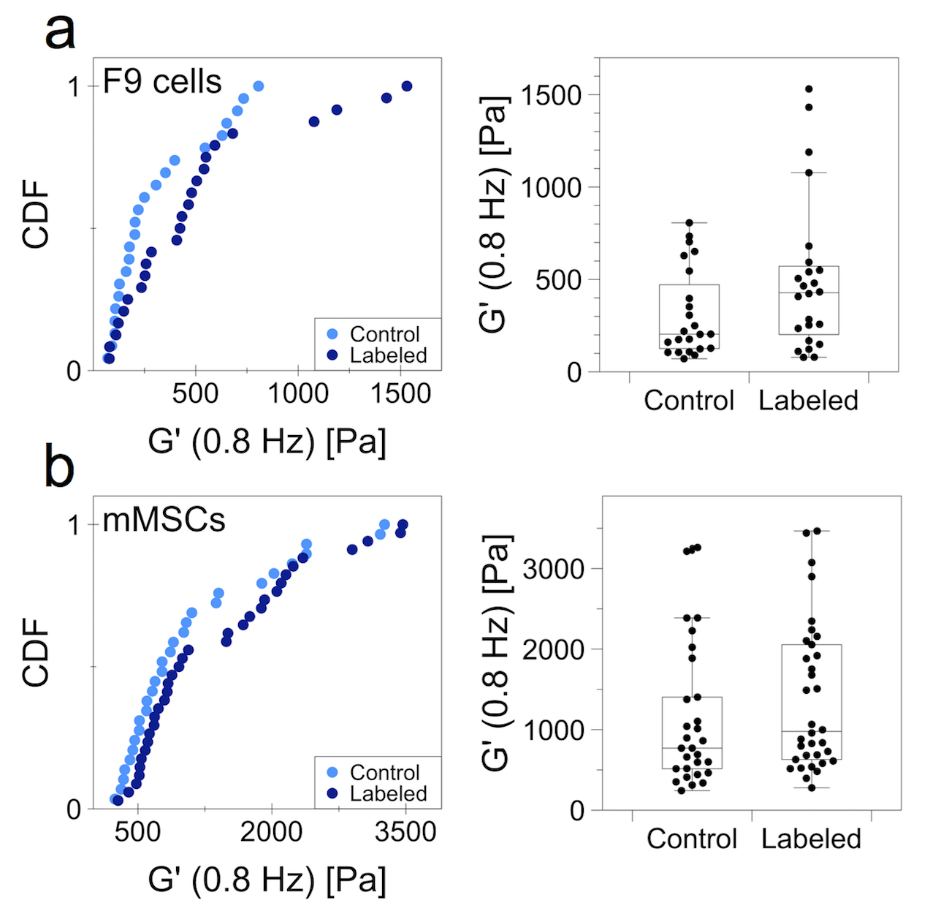


**Figure S2.** Cumulative distribution function (CDF) and box plot representation of G’ (0.8 Hz) for a F9 cells and b mMSCs. (n = 23 for F9 control cells; n = 24 for F9 labeled cells; n = 29 for control mMSCs; n = 34 for labeled mMSCs; *p < 0.05).


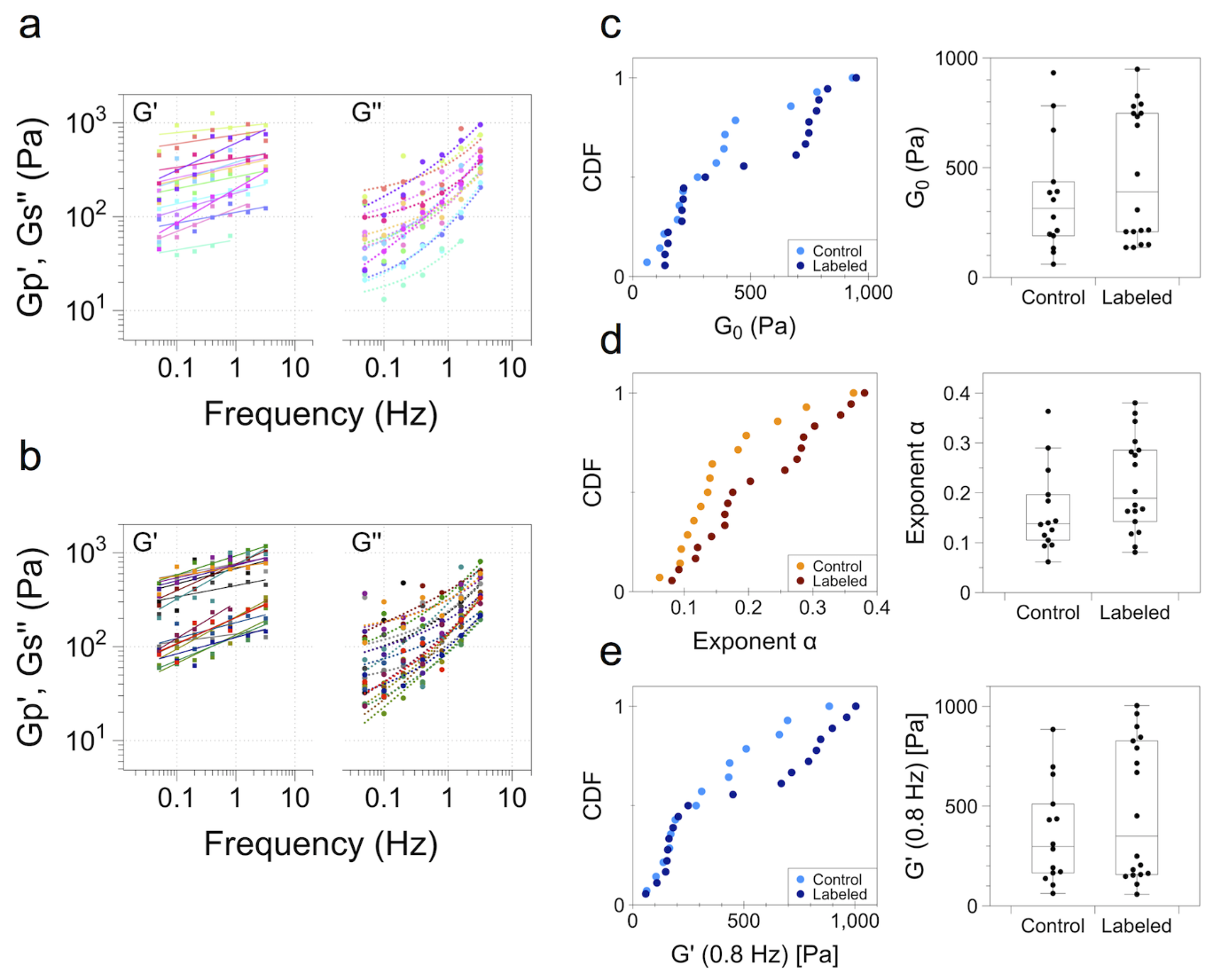


**Figure S3.** Viscoelastic modulus of F9 cells analyzed 24 hours after labeling with magnetic nanoparticles at [Fe] = 4 mM during a 2 hours incubation. **a** and **b** Elastic (*G’*) and viscous (*G’’*) moduli as a function of oscillating stress for control and labeled cells, respectively. **c**, **d** and **e** Cumulative distribution function (CDF) and box plot representation of the prefactor *G_0_*, exponent α and *G’* (0.8 Hz), respectively. (n = 14 for control cells; n = 18 for labeled cells; *p < 0.05).


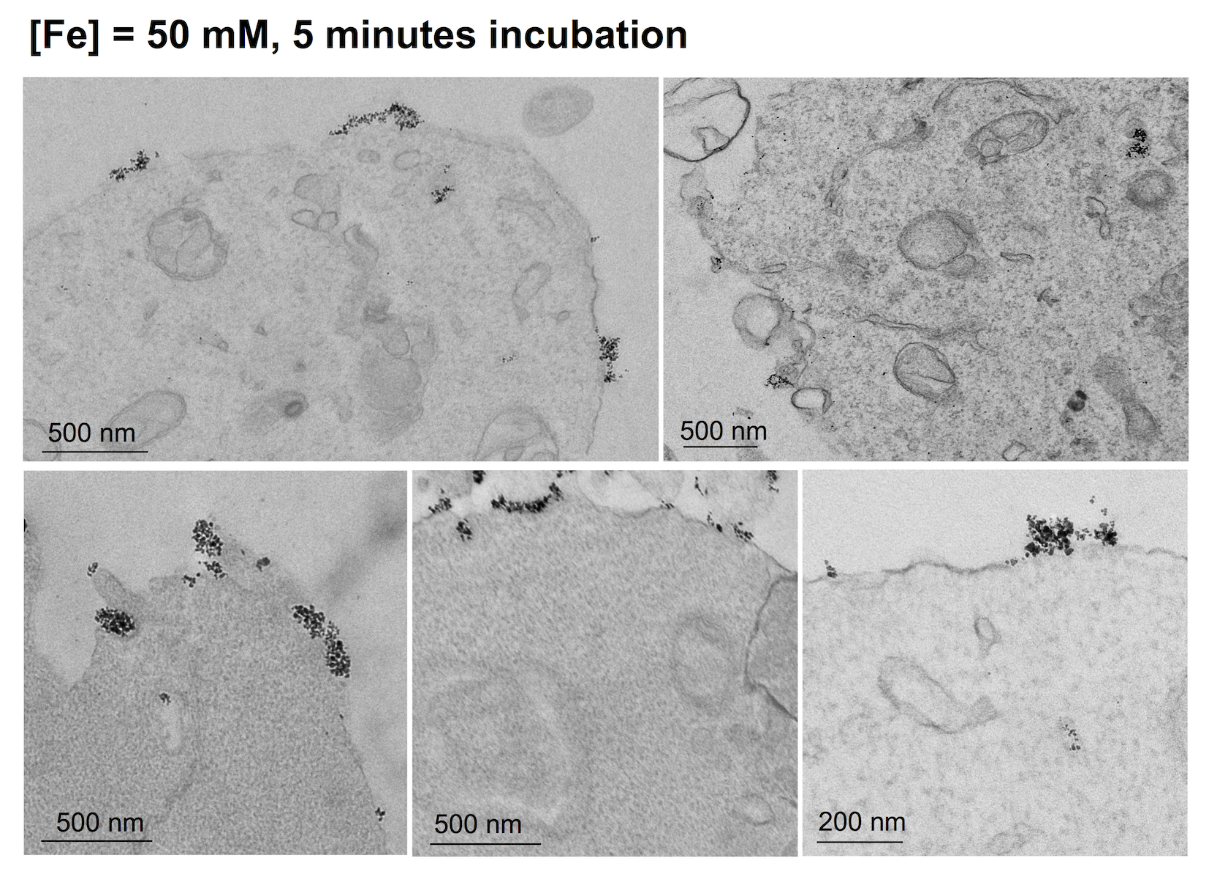


**Figure S4.** TEM imaging of F9 cells labeled with magnetic nanoparticles at [Fe] = 50 mM during a 5 minutes incubation.


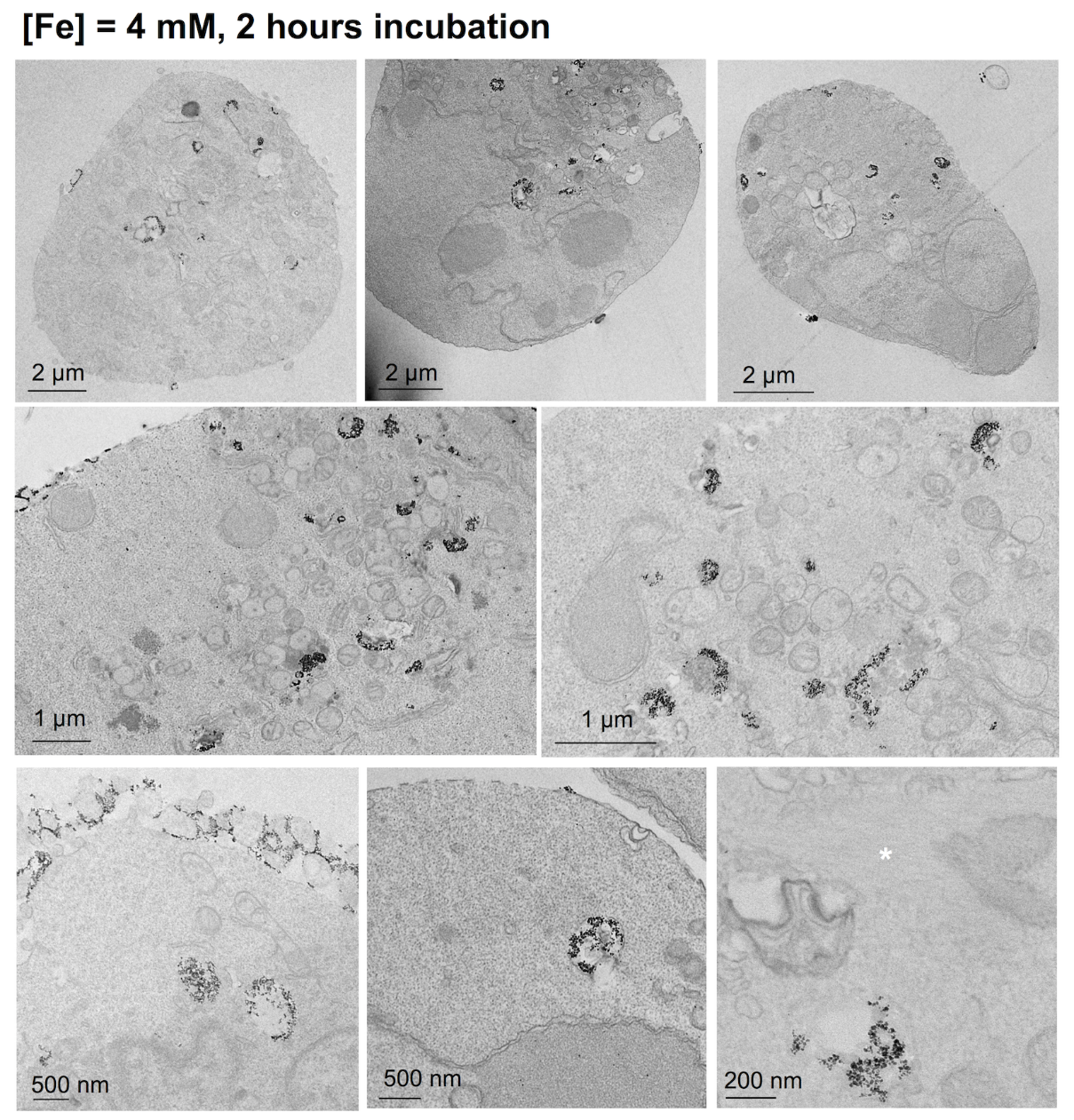


**Figure S5.** TEM imaging of F9 cells labeled with magnetic nanoparticles at [Fe] = 2 mM during a 2 hours incubation.


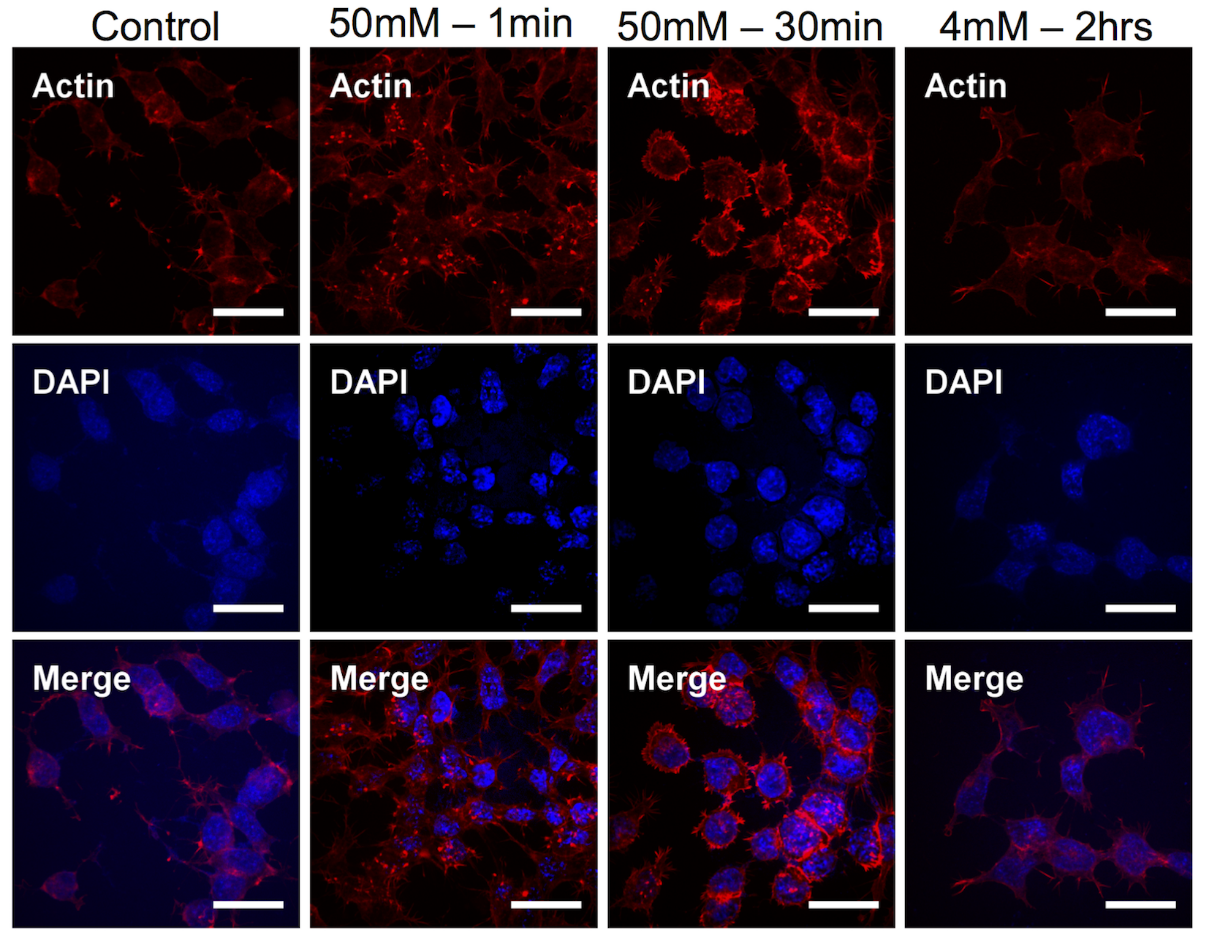


**Figure S6.** Fluorescent staining of actin filaments observed under confocal microscopy. F9 cells incubated with magnetic nanoparticles under three incubation conditions and stained for actin (red) and cell nuclei (DAPI). Scale bars = 30 µm.

**Figure S7.** Evolution of the hydrodynamic size of the nanoparticles when dispersed in RPMI cell medium supplemented with 5 mM of citrate, as measured by Nanosizer (size distribution by intensity). It evidences the good colloidal stability of the nanoparticles. On average, the hydrodynamic diameter equals 49.5 nm initially, and 52.3 and 55.1 nm after 15 and 30 minutes, respectively.
